# *In silico* approach to identify microsatellite candidate biomarkers to differentiate the biovar of *Corynebacterium pseudotuberculosis* genomes

**DOI:** 10.3389/fbinf.2022.931583

**Published:** 2022-09-16

**Authors:** Kenny da Costa Pinheiro, Bruna Verônica Azevedo Gois, Wylerson Guimarães Nogueira, Fabrício Almeida Araújo, Ana Lídia Cavalcante Queiroz, Oscar Cardenas-Alegria, Artur Luiz da Costa da Silva, Antônio Márcio Gomes Martins Júnior, Rommel Thiago Jucá Ramos

**Affiliations:** ^1^ Institute of Biological Sciences, Federal University of Pará, Belém, Pará, Brazil; ^2^ Department of Biochemistry and Immunology, Federal University of Minas Gerais, Belo Horizonte, Minas Gerais, Brazil; ^3^ Laboratory of Genomic and Bioinformatics, Center of Genomics and System Biology, Federal University of Pará, Belém, Pará, Brazil; ^4^ Laboratory of Genetics, Evolution and Bioinformatics, Federal Institute of Pará, Tucuruí, Pará, Brazil

**Keywords:** *Corynebacterium pseudotuberculosis*, caseous lymphadenitis, biomarkers, microsatellites, virulence factors (VFs), simple sequence repeats (SSRs)

## Abstract

*Corynebacterium pseudotuberculosis* is the causative bacterial agent of the zoonotic disease known as caseous lymphadenitis, and it presents several mechanisms of response to host defenses, including the presence of virulence factors (VFs). The genomes of these bacteria have several polymorphic markers known as microsatellites, or simple sequence repeats (SSRs), that can be used to characterize the genome, to study possible polymorphisms existing among strains, and to verify the effects of such polymorphic markers in coding regions and regions associated with VFs. In this study, several SSRs were identified within coding regions throughout the 54 genomes of this species, revealing possible polymorphisms associated with coding regions that could be used as strain-specific or serotype-specific identifiers of *C. pseudotuberculosis*. The similarities associated with SSRs amongst the different serum variants of *C. pseudotuberculosis*, biovars *equi* and *ovis*, were also evaluated, and it was possible to identify SSRs located in coding regions responsible for a VF enrolled in pathogenesis known to mediate bacterial adherence (SpaH-type pili virulence factor). Phylogenetic analyses revealed that strains sharing SSR patterns, including the possible polymorphisms identified in the same position of gene-coding regions, were displayed by strains with a common ancestor, corroborating with the Genome Tree Report of the NCBI. Statistical analysis showed that the microsatellite groups belonging to *equi* and *ovis* biovars have a significance of 0.006 (*p*-value) in similarity, thus indicating them as good biomarker candidates for *C. pseudotuberculosis*.

## Introduction

The intracellular pathogen *Corynebacterium pseudotuberculosis* causes a chronic infectious disease called caseous lymphadenitis, manifested by the presence of necrosis in the lymphatic glands (Radostits et al., 2002). This bacterium has several response mechanisms to host defense, including virulence factors such as the presence of lipids associated with the cell wall that gives the microorganism resistance to digestion by cellular enzymes and, consequently, the ability to spread through the host tissues (Airello et al., 2001).

Studies have already been developed on the genomic structure and virulence of *C. pseudotuberculosis* (Soares et al., 2013; Baraúna et al., 2017; Gomide et al., 2018a; Gomide et al., 2018b; Araújo et al., 2019). Although these studies are extensive, there have been no reports on microsatellites associated with gene composition and virulence in *C. pseudotuberculosis*, and such microsatellites are widely used for genetic studies and as molecular markers (Han et al., 2015). Microsatellites, or simple sequence repeats (SSRs), are found in eukaryotes, prokaryotes, and viruses having a wide distribution throughout the genome, being present in both gene-coding and intergenic regions. SSRs are repeated sequences in tandem, whose repetition unit, called pattern or motif, is between 1 and 10 base pairs long (Jarne and Lagoda, 1996). These tandem repetitions have mutation rates that occur between 10³ and 10^6^ per cell generation, and, due to this instability, have high relevance in evolutionary studies (Vieira et al., 2016).

As SSRs occur throughout the genome of different species, they have become suitable for the study of genetic diversity amongst species and populations. They can be classified according to the type of repetition into the following: (I) perfect microsatellites, presenting perfect repetitions, for e.g., (AT) 20; (II) imperfect microsatellites, presenting interruptions in the repetition caused by different nucleotides than those that occur in the repetitive pattern, for e.g., (AT) 12 GC (AT) 8; and (III) compound microsatellites, containing different motifs (two or more) repeated in tandem, for e.g., (AT) 7 (GC) 6 (Saeed et al., 2016).

Microsatellites are widely used to identify a particular molecular sequence in an unknown DNA pool. Previous studies suggested that the origin of microsatellites in microbial genomes is not random. Several mechanisms can stimulate the presence of SSRs in gene regions, such as insertions, deletions, recombination, transpositions, and horizontal gene transfer (Saeed et al., 2016). These markers are highly polymorphic and can influence gene regulation, thus being used in the studies of kinship and ancestry (Chen et al., 2011).

SSRs are more than just repetitive strings, as they can play an important role in several biological pathways and be inserted within genes responsible for virulence in several pathogenic bacteria. They might also alter the expression of genes involved in the host–pathogen interaction. In addition, the presence of trinucleotide and hexanucleotide repetitions in genes encoding proteins may be highly relevant to the protein 3D structure. In humans, tri- and tetra-motifs located in protein-coding regions are often associated with genetic diseases (Mrázek et al., 2007).

Due to high mutation rates and next-generation sequencing (NGS) technologies, microsatellites are useful molecular markers that can be easily detected by low-cost PCR techniques (Paglia and Morgante, 1998). The screening of SSRs has been poorly explored in certain species of prokaryotes, as prokaryotic genomes are known for containing less repetitive elements in their DNA than those observed in eukaryotes (Metzgar et al., 2001). Therefore, the identification and characterization of SSRs in the genome of the many different strains of *C. pseudotuberculosis* are an important asset in the study of its pathogenicity by identifying possible markers associated with virulence genes.

## Materials and methods

### Genomic dataset

The search for microsatellites in 54 genomes of *Corynebacterium pseudotuberculosis* available in the National Center for Biotechnology Information’s RefSeq database (NCBI) was carried out using the software package of IMEx tools (Mudunuri and Nagarajaram, 2007). The collective strain information of the biovar, host, country, genome size, and number of genes and proteins for each genome used in this study is shown in Table 1. For the scope of this work, we selected only 54 genomes amongst all the genomes available at the time due to the clonal nature of the sequenced strains of *C. pseudotuberculosis* (Soares et al., 2013) and to secure space out of the global dataset, so we would be able to later test our findings from this training dataset on other available non-included strain, using the markers identified by this work.

**TABLE 1:** General information on the genomic dataset of 54 genomes of *Corynebacterium pseudotuberculosis* used in this work.

Species/strain	Biovar	Host	Country	Size (Mb)	Gene	Protein
*C. pseudotuberculosis* I19	*Ovis*	Cow	Israel	2,33821	2,123	2,004
*C. pseudotuberculosis* PAT10	*Ovis*	Sheep	Argentina	2,33830	2,139	1,993
*C. pseudotuberculosis* 267	*Ovis*	llama	USA	2,33790	2,137	2,035
*C. pseudotuberculosis* 226	*Ovis*	Goat	USA	2,33783	2,132	1,966
*C. pseudotuberculosis* 29156	*Ovis*	Cow	Israel	2,33775	2,123	2,006
*C. pseudotuberculosis* PO269-5	*Ovis*	Goat	Portugal	2,33826	2,130	2,010
*C. pseudotuberculosis* 1002B	*Ovis*	Goat	Brazil	2,33831	2,138	2,021
*C. pseudotuberculosis* PA01	*Ovis*	Sheep	Brazil	2,33777	2,138	2,036
*C. pseudotuberculosis* MEX25	*Ovis*	Sheep	Mexico	2,33813	2,132	2,018
*C. pseudotuberculosis* PO222/4-1	*Ovis*	Goat	Portugal	2,33816	2,129	2,014
*C. pseudotuberculosis* E55	*Ovis*	Sheep	Egypt	2,33829	2,126	1,987
*C. pseudotuberculosis* PA02	*Ovis*	Goat	Brazil	2,33834	2,128	2,029
*C. pseudotuberculosis* MEX29	*Ovis*	Sheep	Mexico	2,33780	2,133	2,032
*C. pseudotuberculosis* MEX1	*Ovis*	Goat	Mexico	2,33827	2,134	2,016
*C. pseudotuberculosis* PA04	*Ovis*	Sheep	Brazil	2,33773	2,129	1,982
*C. pseudotuberculosis* PA07	*Ovis*	Sheep	Brazil	2,33820	2,127	1,994
*C. pseudotuberculosis* CAP3W	*Ovis*	Caprine	Brazil	2,33818	2,146	2,028
*C. pseudotuberculosis* CAPJ4	*Ovis*	Caprine	Brazil	2,33808	2,146	2,029
*C. pseudotuberculosis* Cap1W	*Ovis*	Caprine	Brazil	2,33817	2,141	2,024
*C. pseudotuberculosis* CAPMI03	*Ovis*	Caprine	Brazil	2,33812	2,141	2,021
*C. pseudotuberculosis* 04MAT	*Ovis*	Caprine or ovine	Brazil	2,33801	2,141	2,021
*C. pseudotuberculosis* 38MAT	*Ovis*	Caprine or ovine	Brazil	2,33771	2,139	1,992
*C. pseudotuberculosis* OVID04	*Ovis*	Ovine	Brazil	2,33810	2,139	1,995
*C. pseudotuberculosis* OVIOS02	*Ovis*	Ovine	Brazil	2,33793	2,141	2,022
*C. pseudotuberculosis* OVIZ01	*Ovis*	Ovine	Brazil	2,33781	2,139	1,994
*C. pseudotuberculosis* MEX2	*Ovis*	Goat	Mexico	2,33809	2,135	2,015
*C. pseudotuberculosis* PAT16	*Ovis*	Sheep	Argentina	2,33815	2,131	2,014
*C. pseudotuberculosis* PAT14	*Ovis*	Sheep	Argentina	2,33825	2,129	2,008
*C. pseudotuberculosis* CIP 52.97	*Equi*	Horse	Kenya	2,33748	2,164	2,039
*C. pseudotuberculosis* 1/06-A	*Equi*	Horse	USA	2,33835	2,101	1,863
*C. pseudotuberculosis* 31	*Equi*	Buffalo	Egypt	2,33727	2,204	2,058
*C. pseudotuberculosis* 258	*Equi*	Horse	Belgium	2,33749	2,164	2,037
*C. pseudotuberculosis* Cp162	*Equi*	Camel	UK	2,33736	2,162	2,009
*C. pseudotuberculosis* 262	*Equi*	Cow	Belgium	2,33757	2,156	2,032
*C. pseudotuberculosis* E19	*Equi*	Horse	Chile	2,33753	2,179	2,043
*C. pseudotuberculosis* MB11	*Equi*	Horse	USA	2,33741	2,167	2,027
*C. pseudotuberculosis* MB14	*Equi*	Horse	USA	2,33740	2,176	1,962
*C. pseudotuberculosis* MB30	*Equi*	Horse	USA	2,33752	2,171	2,026
*C. pseudotuberculosis* MB66	*Equi*	Horse	USA	2,33737	2,175	1,955
*C. pseudotuberculosis* MB20	*Equi*	Horse	USA	2,33739	2,180	1,896
*C. pseudotuberculosis* 32	*Equi*	Buffalo	Egypt	2,33730	2,216	2,077
*C. pseudotuberculosis* 33	*Equi*	Buffalo	Egypt	2,33729	2,214	2,072
*C. pseudotuberculosis* 34	*Equi*	Buffalo	Egypt	2,33733	2,212	2,076
*C. pseudotuberculosis* 35	*Equi*	Buffalo	Egypt	2,33732	2,216	2,074
*C. pseudotuberculosis* 36	*Equi*	Buffalo	Egypt	2,33734	2,211	2,068
*C. pseudotuberculosis* 38	*Equi*	Buffalo	Egypt	2,33731	2,210	2,065
*C. pseudotuberculosis* 39	*Equi*	Buffalo	Egypt	2,33728	2,209	2,070
*C. pseudotuberculosis* 43	*Equi*	Buffalo	Egypt	2,33756	2,170	2,037
*C. pseudotuberculosis* 46	*Equi*	Buffalo	Egypt	2,33755	2,167	2,034
*C. pseudotuberculosis* 48	*Equi*	Buffalo	Egypt	2,33735	2,211	2,072
*C. pseudotuberculosis* I37	*Equi*	Cow	Israel	2,33742	2,166	2,029
*C. pseudotuberculosis* MEX30	*Equi*	Horse	Mexico	2,33751	2,173	2,010
*C. pseudotuberculosis* MEX31	*Equi*	Horse	Mexico	2,33754	2,182	2,058
*C. pseudotuberculosis* 316	*Equi*	Horse	USA	2,33750	2,162	2,025

### Simple sequence repeat identification

IMEx software identified the perfect microsatellites for the genome of each of the 54 selected strains, in the form of nucleotide sequences. The tool also accepts.ptt (Protein Table File) files as input, allowing the identification of SSRs located in genetic and intergenic regions. We established the perfect microsatellite search parameters in the IMEx tool as follows (size of motifs—the minimum number of repetitions): 1-12, 2-6, 3-4, 4-3, 5-3, and 6-3; based on research by Chen et al. (2011). The online tool VFanalyzer (Liu et al., 2019) was used to identify virulence factors (VFs) in the 54 genomes, and then only VFs containing microsatellites inserted in their sequences were selected.

### Assessment of SSR patterns

The WEB BedSect tool (Mishra et al., 2020) was used to evaluate all SSRs regarding their positions in the genome to identify all possible similarities among the 54 genomes in this study. Additionally, two extra genomes of the same species were used for a biovar identification test by the position and type of microsatellite detected, considering the profile patterns discovered in this study. The selected genomes were *C. pseudotuberculosis* C231 belonging to the *ovis* biovar and *C. pseudotuberculosis* MB154 from the *equi* biovar.

### Visualization of data

The results presented in the form of bar graphs were generated by the statistical analysis software environment R (https://www.r-project.org/) (R.D.C.T. 3.5.1, 2018). The output of the WEB BedSect tool was presented in the form of a heatmap. All bar graphs associated with each genome individually not presented in the discussion are provided in the Supplementary Material. The visualization of annotation and sequence features was executed using the Artemis genome browser (Carver et al., 2012).

### Dataset for phylogenetic analysis

We conducted a phylogenetic analysis to check whether the heatmap and clusters recovered by the WEB BedSect tool match phylogenetic groups. A dataset formed by 38 genes that contain microsatellites shared by all 54 studied genomes was produced. For this, each gene for every sample of the 54 genomes was individually aligned and checked visually using MUSCLE software (Edgar, 2004), a plug-in from PhyDE^®^ software (Müller et al., 2006). All aligned genes were concatenated using SequenceMatrix 1.8 software (Vaidya et al., 2011) to produce a dataset with 42,606 bp length, which is available in Supplementary Appendix S1.

### Evolutionary model and partition scheme selection

PartitionFinder 2.1.1 software (Lanfear et al., 2017) was used to find the best-fit partitioning scheme of the dataset and the evolutionary nucleotide substitution model for each partition. All genes were defined according to the codon positions. The rcluster searching method was used to test all models implemented using RAxML 8.2.10 software (Stamatakis, 2014). The best models were selected by AICc values. Complete information on the partitioning schemes and the evolutionary nucleotide substitution model selected for each partition is presented in Supplementary Table S1.

### Phylogenetic analysis

Phylogenetic reconstructions among different lineages of *Corynebacterium pseudotuberculosis* were performed using the maximum likelihood (ML) and neighbor-joining (NJ) algorithms. The ML analysis was carried out in RAxML 8.2.10 software (Stamatakis, 2014) using the selected partitioning schemes and substitution models (Supplementary Table S1). The most likelihood tree was searched 1,000 times, and the support of the internal nodes was estimated by 1,000 pseudo-replicates of bootstrap. The NJ analysis was carried out in MEGA X software (Kumar et al., 2018) using the K2P + G substitution model. Node support was estimated by bootstrap, using 1,000 pseudo-replicates.

### Statistical analysis

The full dataset of microsatellites from the *ovis* biovar was gathered and imported into the RStudio software environment using the universal motif package (RStudio Team, 2020; Tremblay, 2022). The same step was performed for the microsatellites of *equi* biovar. Using the functions of the universal motif package, we merged all the motifs into two separate categories (*ovis* and *equi*). We applied a Euclidean distance method between the two groups to measure their similarity and represent the two microsatellite categories through an information content matrix. This calculation is based on Shannon’s entropy (Shannon, 1948), with the final values representing “bits” (Schneider, 1991).

## Results

**FIGURE 1:**
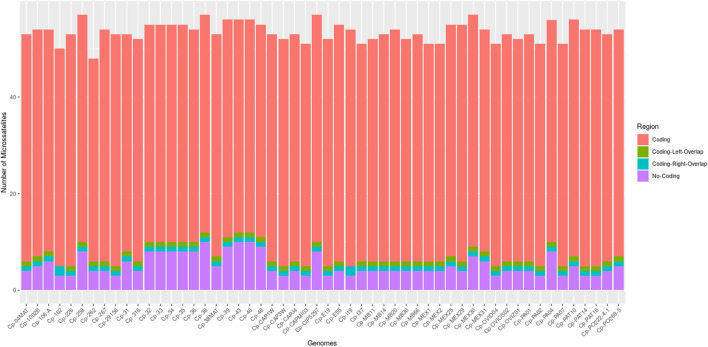
Bar graph showing the number of microsatellites found in the 54 genomes of *C. pseudotuberculosis*, divided into four categories: coding, non-coding, coding left overlap, and coding right overlap.

Through the IMEx tool, it was possible to locate all microsatellites fully inserted in coding regions, in non-coding regions, partially inserted in coding regions on the left (coding left overlap), and partially inserted in coding regions on the right (coding right overlap) (Figure 1). All genomes had microsatellites inserted in these four categories, except for the genomes of *C. pseudotuberculosis* 162 and *C. pseudotuberculosis* I19 that did not present microsatellites partially inserted in coding regions on the left (coding left overlap).

The genomes with the highest amount of SSRs were *C. pseudotuberculosis* MEX30, *C. pseudotuberculosis* CIP 52.97, *C. pseudotuberculosis* 38, and *C. pseudotuberculosis* 258, with a count of 57 SSRs each. All the other genomes in the study had a count of fewer than 57 microsatellites (Supplementary Table S1).

**FIGURE 2:**
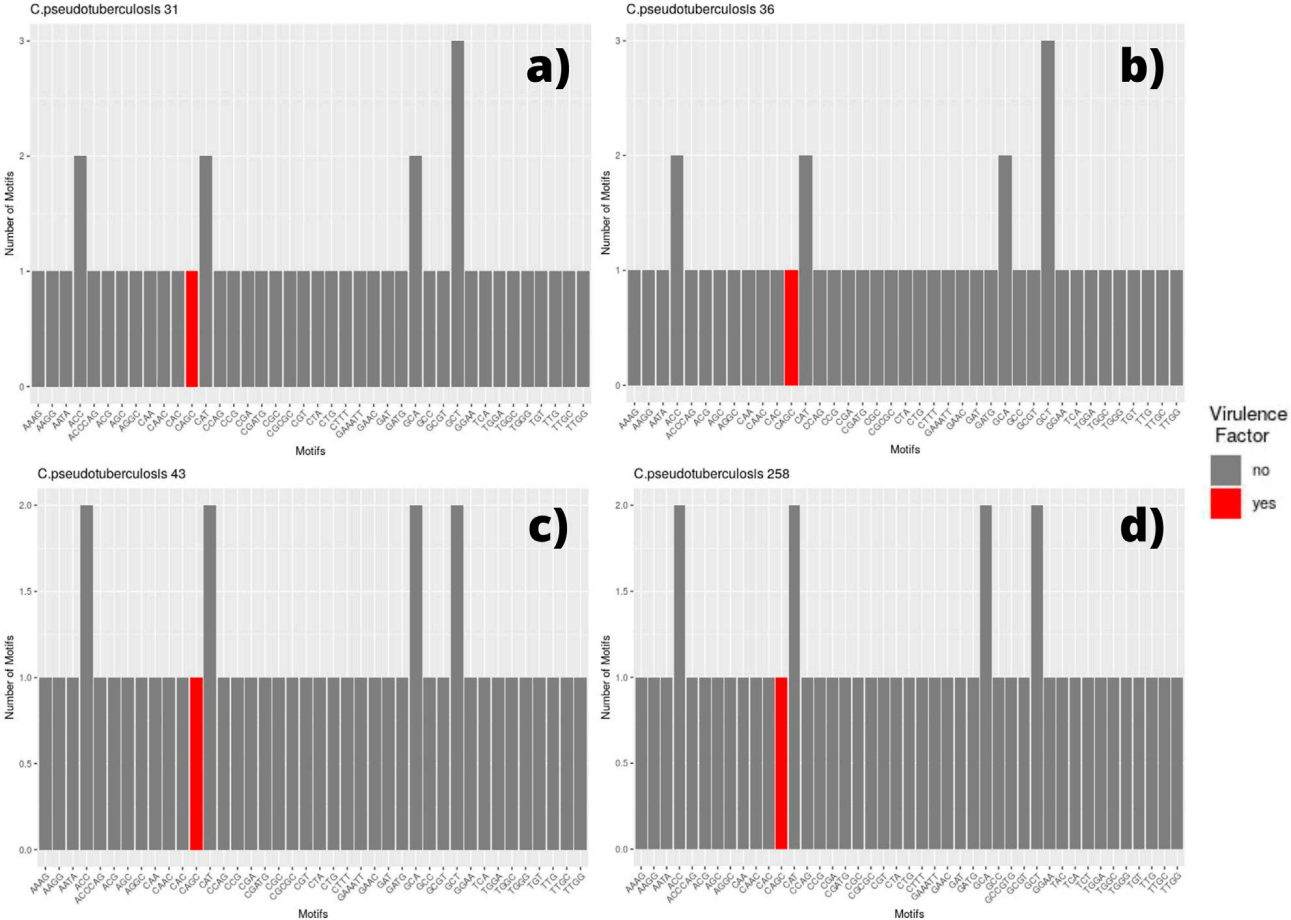
Bar graph showing the number of different SSR motifs observed in *Corynebacterium pseudotuberculosis* strains. In order, strain 31 **(A)**; strain 36, with remarks to the absence of the CGT motif in this genome **(B)**; strain 43, with remarks to the GCT motif occurring twice in this genome in different regions **(C)**; and strain 258, presenting the same pattern that can be observed in *C. pseudotuberculosis* CIP 52.97 **(D)**.

**FIGURE 3:**
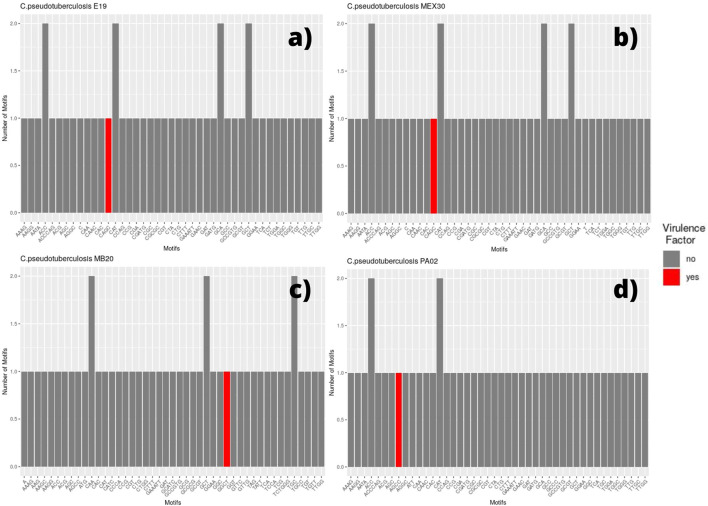
Bar graph showing the number of different SSR motifs observed in *Corynebacterium pseudotuberculosis* strains. In order, strain E19 **(A)**; strain MEX 30 **(B)**; strain MB20, in which the microsatellite with a GGCT motif occurs within a virulence factor located on the direct ribbon **(C)**; and strain PA02, in which the microsatellite with an AGCC motif occurs within a virulence factor located on the reverse ribbon **(D)**.

We selected every SSR present in coding regions and screened for the presence of those same motifs in different genomic regions. Thus, for each genome, a bar plot was made to display the frequency of these motifs. Therefore, if a motif has a frequency equal to two, it means that we can find the same motif in two different coding regions (two different genes with the same motif). The bar plot also displays the motif occurring within a coding region for a virulence factor, which is marked in red (Figures 2, 3).

### Biovar *equi*

Among the genomes belonging to the *equi* biovar, we identified that the strains *C. pseudotuberculosis* 31, 32, 33, 34, 35, 36, 38, and 39 presented the same pattern, which can be observed in Figure 2, which shows the genome of *C pseudotuberculosis* 31 (Figure 2A). The presence of a motif located within a gene encoding for a virulence factor, the motif CAGC, is highlighted in the plot by a red bar.

In this genome, we can see that the most frequent motifs are: GCT, present in three different coding regions—hypothetical protein, cation-translocating P-type ATPase, and transporter; ACC, present in two different coding regions—elongation factor G and SPFH/Band 7/PHB domain protein; CAT, present in two distinct coding regions—potassium channel family protein and GNAT family N-acetyltransferase; and GCA, also present in two distinct regions—DNA-binding protein WhiA and S8 family peptidase. The only difference observed between these genomes (*C. pseudotuberculosis* 31, 32, 33, 34, 35, 36, 38, and 39) occurred in the genome of *C. Pseudotuberculosis* 36 in which the CGT motif was not found (Figure 2B).

The strains *C. pseudotuberculosis* 43 (Figure 2C) showed similar SSR patterns to *C. pseudotuberculosis* 46 and 31 strains, except for the GCT motif, presented in three distinct coding regions for *C. pseudotuberculosis* 31 and only in two distinct regions for *C. pseudotuberculosis* 43 and 46 strains. The *C. pseudotuberculosis* 258 (Figure 2D) and CIP 52.97 strains also showed identical microsatellite profiles from their genomes.

The SSR profile of *C. pseudotuberculosis* E19 was similar to strains 258 and CIP 52.97, except for a single microsatellite having a mononucleotide C as a motif inserted in a gene encoding the NADP-dependent oxidoreductase product (Figure 3A). Single microsatellites of mononucleotide C and mononucleotide T were also observed in the genome of *C. pseudotuberculosis* MEX 30 (Figure 3B).

On the *equi* biovar, the presence of a microsatellite inserted within a virulence factor coding region was observed at every genome. The inserted SSR for most of the strains presented a CAGC motif, whereas, for *C. pseudotuberculosis* MB20 and MB66 strains, it was a GGCT motif (Figure 3C).

### Biovar *ovis*

Regarding the genomes of *ovis* biovar, the *C. pseudotuberculosis* strains 226, 267, 29156, I19, MEX25, MEX29, PAT10, PAT14, and PAT16 did not present any microsatellites inserted within genes that encode virulence factors (Supplementary Figure S1). Still, while in the *equi* biovar the CAGC motif was identified in most of the VFs found in each genome; in the *ovis* biovar, the most identified motif was GGCT (Supplementary Figure S1), except for the *C. pseudotuberculosis* PA01, PA04, and PA07 strains in which the observed motif was CAGC (Supplementary Figure S1). Remarkably, it was observed that in the genome of *C. pseudotuberculosis* PA02, unlike all genomes in this study, the motif associated with the virulence factor was AGCC (Figure 3D).

### WEB BedSect analysis

**FIGURE 4:**
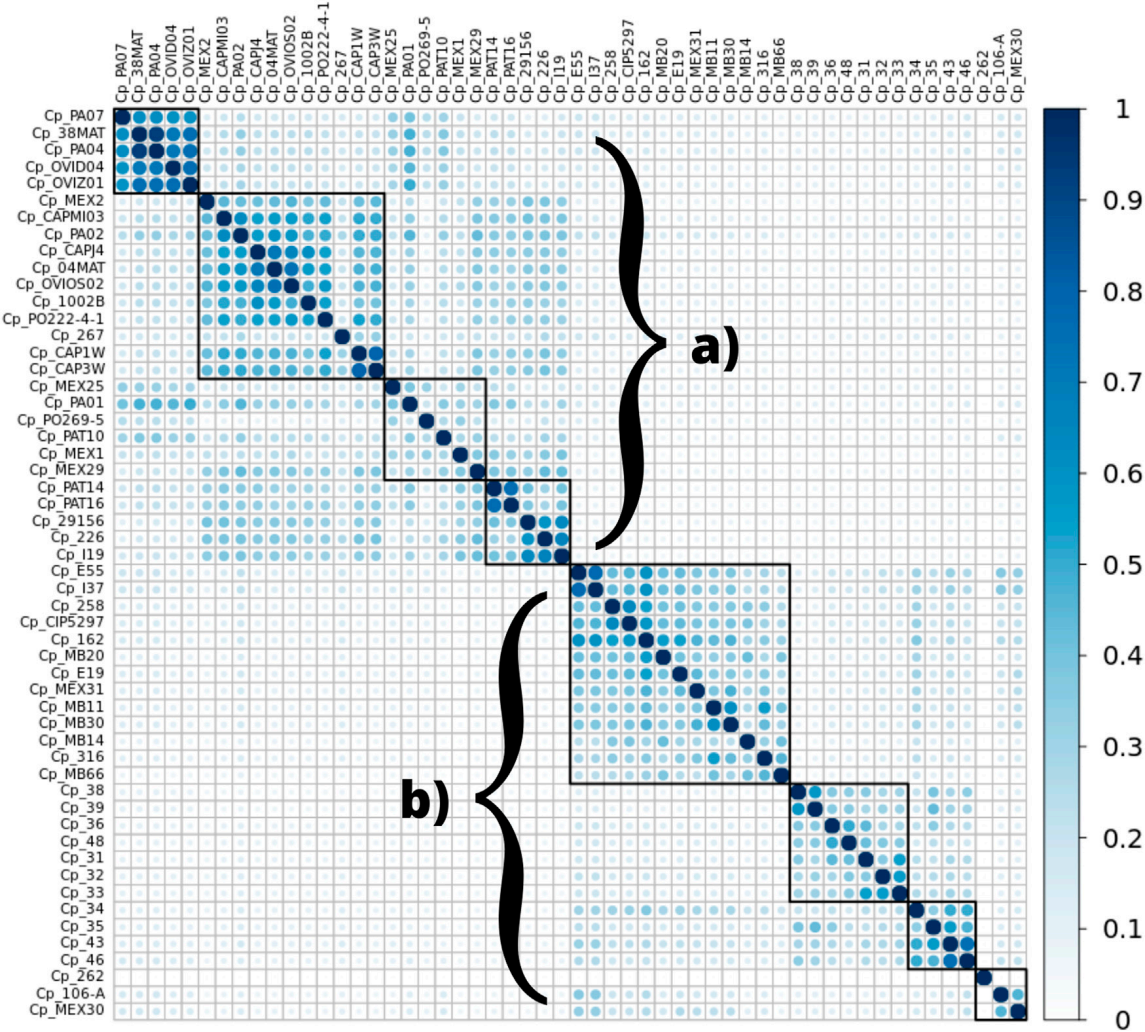
Heatmap demonstrating the similarity between microsatellite regions among the 54 genomes of *C. pseudotuberculosis* in this study, in particular displaying a significant separation between biovars *ovis* **(A)** and *equi* **(B)**. The similarity observed is displayed on a color scale from blank to light blue to darker blue (a greater number of identical regions).

The analysis of the genomic regions containing microsatellites showed similarity among the genomes of the *ovis* biovar, and they displayed an intrinsic similarity among the genomes from the *equi* biovar. It was possible to distinguish the two biovars of *C. pseudotuberculosis* by analyzing the patterns observed inter-biovars (*ovis* vs. *equi*) and intra-biovars (*ovis* vs. *ovis*, and *equi* vs. *equi*), comparing exclusively the regions where SSRs were found for every genome of this study. The following graph shows a remarkable separation between biovars (Figure 4).

Some microsatellites identified by the WEB BedSect intersections were selected to identify the differences between the *ovis* and *equi* biovars. There was a positional change in these motifs occurring between biovars (Tables 2, 3).

**TABLE 2:** Localization of the GGAA motif between *equi* and *ovis* biovars.

Motif	Start	End	Biovar	Motif	Start	End	Biovar	Motif	Start	End	Biovar
GGAA	42,622	42,633	*Ovis*	GGAA	44,639	44,640	*Ovis*	GGAA	52,758	52,769	*Equi*
GGAA	44,251	44,262	*Ovis*	GGAA	44,640	44,641	*Ovis*	GGAA	52,800	52,810	*Equi*
GGAA	44,621	44,623	*Ovis*	GGAA	44,641	44,642	*Ovis*	GGAA	52,810	52,811	*Equi*
GGAA	44,623	44,626	*Ovis*	GGAA	44,642	44,643	*Ovis*	GGAA	52,811	52,814	*Equi*
GGAA	44,626	44,627	*Ovis*	GGAA	52,021	52,022	*Equi*	GGAA	52,814	52,818	*Equi*
GGAA	44,627	44,628	*Ovis*	GGAA	52,022	52,023	*Equi*	GGAA	52,818	52,821	*Equi*
GGAA	44,628	44,629	*Ovis*	GGAA	52,023	52,024	*Equi*	GGAA	52,821	52,825	*Equi*
GGAA	44,629	44,630	*Ovis*	GGAA	52,024	52,026	*Equi*	GGAA	52,825	52,829	*Equi*
GGAA	44,630	44,631	*Ovis*	GGAA	52,026	52,032	*Equi*	GGAA	52,829	52,832	*Equi*
GGAA	44,631	44,632	*Ovis*	GGAA	52,032	52,033	*Equi*	GGAA	52,832	52,836	*Equi*
GGAA	44,632	44,634	*Ovis*	GGAA	52,033	52,034	*Equi*	GGAA	52,836	52,847	*Equi*
GGAA	44,634	44,637	*Ovis*	GGAA	52,034	52,035	*Equi*	GGAA	56,279	56,290	*Equi*
GGAA	44,637	44,638	*Ovis*	GGAA	52,035	52,037	*Equi*	GGAA	57,146	57,157	*Equi*
GGAA	44,638	44,639	*Ovis*	GGAA	52,698	52,709	*Equi*	GGAA	57,167	57,178	*Equi*

**TABLE 3:** Localization of the CAC motif between *equi* and *ovis* biovars.

Motif	Start	End	Biovar	Motif	Start	End	Biovar	Motif	Start	End	Biovar
CAC	54,914	54,925	*Ovis*	CAC	56,951	56,952	*Ovis*	CAC	65,108	65,119	*Equi*
CAC	56,564	56,575	*Ovis*	CAC	56,952	56,953	*Ovis*	CAC	65,119	65,125	*Equi*
CAC	56,905	56,916	*Ovis*	CAC	56,953	56,954	*Ovis*	CAC	65,125	65,129	*Equi*
CAC	56,932	56,936	*Ovis*	CAC	56,954	56,955	*Ovis*	CAC	65,129	65,130	*Equi*
CAC	56,936	56,938	*Ovis*	CAC	56,955	56,956	*Ovis*	CAC	65,130	65,134	*Equi*
CAC	56,938	56,939	*Ovis*	CAC	64,331	64,332	*Equi*	CAC	65,134	65,136	*Equi*
CAC	56,939	56,940	*Ovis*	CAC	64,332	64,333	*Equi*	CAC	65,136	65,138	*Equi*
CAC	56,940	56,941	*Ovis*	CAC	64,333	64,334	*Equi*	CAC	65,138	65,140	*Equi*
CAC	56,941	56,942	*Ovis*	CAC	64,334	64,336	*Equi*	CAC	65,140	65,145	*Equi*
CAC	56,942	56,943	*Ovis*	CAC	64,336	64,342	*Equi*	CAC	65,145	65,147	*Equi*
CAC	56,943	56,944	*Ovis*	CAC	64,342	64,343	*Equi*	CAC	65,147	65,149	*Equi*
CAC	56,944	56,945	*Ovis*	CAC	64,343	64,344	*Equi*	CAC	65,149	65,158	*Equi*
CAC	56,945	56,947	*Ovis*	CAC	64,344	64,345	*Equi*	CAC	66,032	66,043	*Equi*
CAC	56,947	56,949	*Ovis*	CAC	64,345	64,347	*Equi*	CAC	69,456	69,467	*Equi*
CAC	56,949	56,950	*Ovis*	CAC	65,009	65,020	*Equi*	CAC	69,477	69,488	*Equi*
CAC	56,950	56,951	*Ovis*	CAC	65,068	65,079	*Equi*				

The GGAA motif always appears between coordinates 42,000 and 45,000 in the *ovis* biovar, whereas, in the *equi* biovar, it occurs between coordinates 52,000 and 57,000, approximately. Likewise, the CAC motif always appears between the coordinates 54,000 and 56,900 in the *ovis* biovar, whereas, in the *equi* biovar, it occurs approximately between coordinates 64,000 and 69,000. This positional difference of microsatellites between biovars was also observed for the CAA, CTG, TGT, and CTG motifs, among others, making these microsatellites potential biomarkers capable of distinguishing biovars.

To test this potential, two genomes (*C. pseudotuberculosis* C231—*ovis* and *C. pseudotuberculosis* MB154—*equi*) that were not amongst the 54 initial genomes in this study were selected, and the CAC and GGAA motifs were used to identify the biovars by the position and type of microsatellite observed. Thus, other strains out of the scope of this work had their biovars successfully identified only by the coordinates of SSRs found in this study (Table 4).

**TABLE 4:** Location of CAC and GGAA motifs for a test of biovar identification.

Genome	CAC	GGAA	Biovar
*C. pseudotuberculosis* C231	56941–56952	44628–44639	*Ovis*
*C. pseudotuberculosis* MB154	65124–65135	52813–52824	*Equi*

### Phylogenetic analysis

**FIGURE 5:**
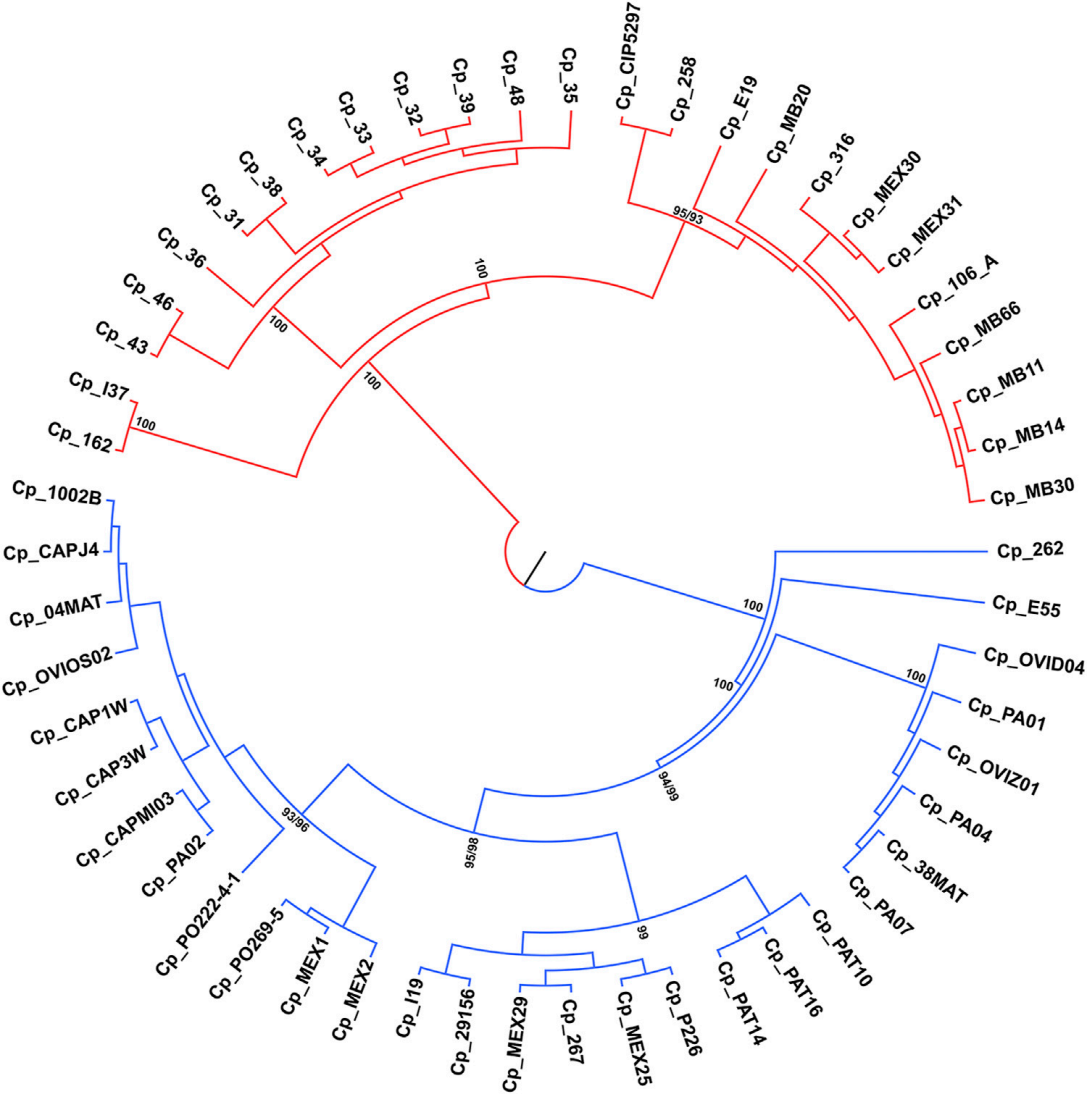
Phylogenetic tree reconstruction of the 54 genomes of *C. pseudotuberculosis*, branching into two main clades with maximum bootstrap values for the *ovis* (in blue) and *equi* (in red) biovars.

ML and NJ tree reconstructions produced similar branching patterning. Two main clades were obtained with maximum bootstrap values, which reflect the *ovis* and *equi* groups (Figure 5) with one exception. The sample Cp_262, which is an *equi* lineage, was more similar to the *ovis* samples, grouping with them in the basal position of this clade (Figure 5). To investigate this incongruence, we compute the pairwise genetic distances between all samples using *p*-distance. As shown in Supplementary Table S2, Cp_262 is slightly more similar to the *ovis* samples (*p*-distance from 0.66 to 0.68%) than to the *equi* ones (*p*-distance from 0.82 to 0.91%). However, the genetic divergences between almost all samples of *ovis* were less than 0.09%, except for the sample Cp_267, which ranged from 0.13 to 0.18% (Supplementary Table S2). It shows that Cp_262 is different from both *ovis* and *equi* clades. Three monophyletic groups were recovered in each *ovis* clade and *equi* clade from both ML and NJ analyses, but the phylogenetic relationships within each group were inconsistent (Figure 5, Supplementary Figure S1).

### Statistical analysis

**FIGURE 6:**
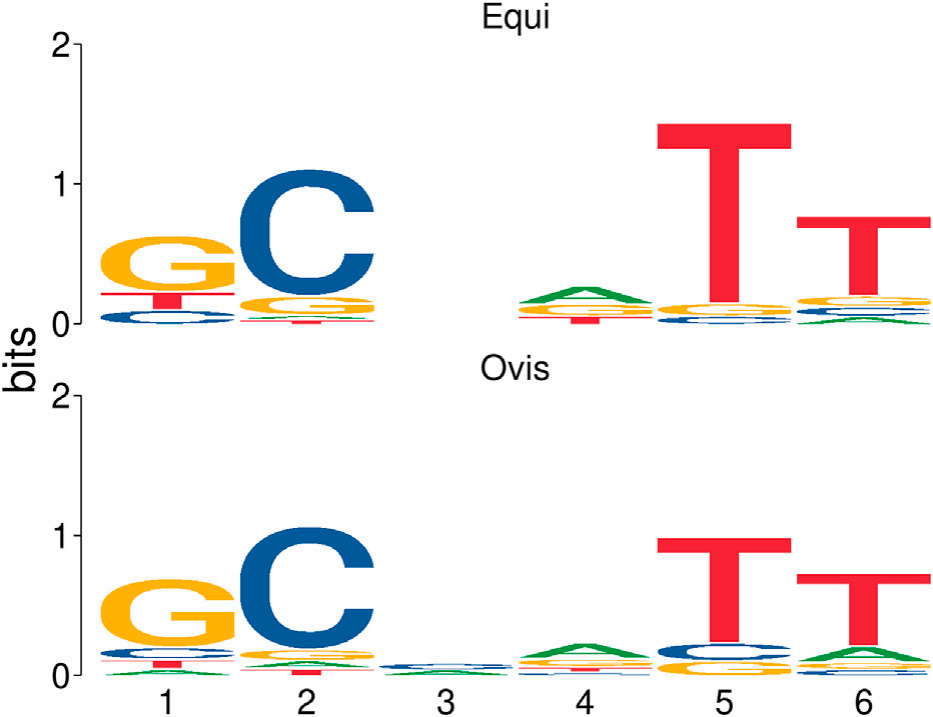
Sequence logo representation based on an information content matrix comparison between *ovis* and *equi* microsatellite biovars. In this figure, we can see the difference in position 3 and the similarities in the other positions when comparing the two sets of microsatellites by using the information content matrix method.

The two groups of microsatellites, *equi* and *ovis* biovars, were similar under statistical analysis, presenting a Euclidean distance score of 0.169405 and a significance of 0.006 (*p*-value). The probability of each base for each microsatellite position observed was estimated. These results are represented through an information content matrix where it is possible to evaluate which positions are the most important, as each position’s total information indicates the conservation level. Thus, we can graphically observe this matrix in the form of a sequence logo, highlighting a difference between the *equi* and *ovis* biovars in position 3, indicating the possibility of using them as markers for the different biovars (Figure 6).

## Discussion

Microsatellites are repetitive elements characterized by having a high degree of polymorphism, hence, less likely to be evolutionarily retained in essential gene clusters (Oliveira et al., 2006). According to the results observed in Figure 1, the amount of SSRs observed in coding regions of *Corynebacterium pseudotuberculosis* is much higher than that in non-coding regions. This observation could be understood as a result of prokaryotic genomes having a smaller number of intergenic regions than eukaryotic genomes (Koonin and Wolf, 2008).

The only virulence factor that contained SSRs observed in the genomes of this study is associated with a structure known as SpaH-type pili. A 2007 study on *Corynebacterium diphtheriae* reported that the low conservation rate of this SpaH locus suggests that mutations in these regions are better tolerated because they are less important for *C. diphtheriae* infection. Adherence tests have shown that the pili of the SpaH type preferentially mediate binding to the cells of the larynx and lung (Mandlik et al., 2007). The presence of SSRs occurring only in this VF in *C. pseudotuberculosis* suggests that it is also well-tolerated by this species and possible polymorphisms in such regions would not affect its virulence. However, further *in vitro* studies of *C. pseudotuberculosis* would be necessary to confirm this hypothesis.

**FIGURE 7:**
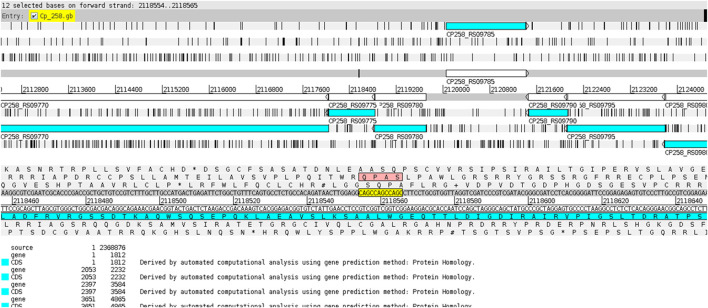
Display of the CAGC motif inserted within the virulence factor SpaH on the reverse ribbon of the genome of *C. pseudotuberculosis* 258 using the Artemis genome browser (Carver et al., 2012).

**FIGURE 8:**
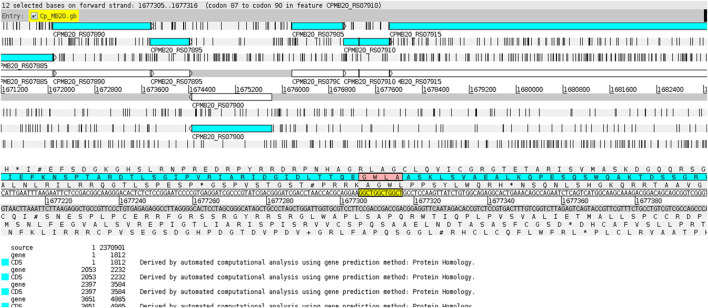
Display of the GGCT motif inserted within the virulence factor SpaH on the direct ribbon of the genome of *C. pseudotuberculosis* MB20 using the Artemis genome browser (Carver et al., 2012).

The CAGC motif inserted within the VF-coding region occurred whenever the gene was located in the reverse strand (Figure 7), while the GGCT motif was found in the VF when the corresponding gene was on the direct strand (Figure 8). This fact explains why the strains MB20 (Figure 8) and MB66 were the only ones in the *equi* biovar to present the GGCT motif, as, unlike the others of this biovar, the virulence factor observed in these two strains was on the direct strand. Therefore, such results suggest a potential for using different microsatellites located in the same gene to identify positional changes associated with the DNA strand of these genes in different genomes of *C. pseudotuberculosis*.

Some genomes of the *equi* biovar (*C. pseudotuberculosis* 31, 32, 33, 34, 35, 36, 38, and 39) showed 100% similarity in all microsatellite regions observed, except for the *C. pseudotuberculosis* 36 strain in which the CGT motif was not found (Figure 2B; Supplementary Table S2). According to the phylogenetic analyses, the *p*-distance also showed that these genomes are 100% equal, including strains 36 and 48 (Supplementary Table S2). The samples of *C. pseudotuberculosis* 43 and 46 differ from the other samples only by 0.0023%. This CGT motif was found in a gene that encodes an HNN endonuclease (HNHE), encoded by many bacteriophage and prophage genomes next to their cohesive end site and terminase genes (Xu and Gupta, 2013).

While looking at the flanking regions in the genomes, the following arrangement of bases was detected: TGG CGT CGT CGT CGT GAA. However, in the genome of *C. pseudotuberculosis* 36, the arrangement of bases was as follows: TGG CGT CGT CGT GAA. Hence, it was not identified as an SSR because a tandem repetition of at least four times is required to identify a trinucleotide motif. This suggests a possible polymorphism in this region of *C. pseudotuberculosis* 36, where a CGT trinucleotide was deleted. The same SSR pattern displayed by strain 36 can be seen in strain 48, including the possible polymorphism identified in the same position of the gene encoding for an HNNE. This result corroborates with the Genome Tree Report of the NCBI (Agarwala et al., 2018), as well as with phylogenetic reconstructions (Figure 5) where these two strains (*C. pseudotuberculosis* 36 and *C. pseudotuberculosis* 48) have a common ancestor.

The *C. pseudotuberculosis* 43 (Figure 2C) and 46 strains also showed patterns of microsatellites identical to each other and similar to *C. pseudotuberculosis* 31, while strains 43 and 46 have an ancestor in common, corroborated by the Genome Tree Report of the NCBI (Agarwala et al., 2018) and phylogenetic trees (Figure 5). Identical microsatellites can also be observed in the *C. pseudotuberculosis* 258 and CIP 52.97 strains, which are genetically identical to each other (Supplementary Table S2) and share a common ancestor (Figure 5). Interestingly, the *C. pseudotuberculosis* MEX30 strain was the only one from the *equi* biovar to present in its genome two mononucleotides as microsatellites. Such characteristics demonstrate the potential use of these microsatellites as biomarkers capable of differentiating strains of the same species.

Regarding the genomes of the *ovis* biovar, some strains (*C. pseudotuberculosis* 226, 267, 29156, I19, MEX25, MEX29, PAT10, PAT14, and PAT16) did not show the virulence factor SpaH and, consequently, did not show any microsatellites with GGCT or CAGC motifs, which are the ones that occur within this VF, once again showing the potential of using such SSRs to screen for possible changes in the genome as well as for the absence of some genes.

The major difference between the motifs found in VFs occurred in the genome of *C. pseudotuberculosis* PA02, where the observed motif within the *SpaH* gene was AGCC (Figure 3D). This fact can be explained by a polymorphism that may have occurred in this region (2080272–2080287 bases) of the genome of *C. pseudotuberculosis* PA02 (TTGG AGCC AGCC AGCC AGCC TT), where the change from cytosine to guanine (highlighted in red) changed the pattern of tandem nucleotides, as seen in the strains PA01, PA04, and PA07 (TTGG AGG CAGC CAGC CAGC CTT).

The *C. pseudotuberculosis* C231 strain presented the CAC motif at coordinates 56941–56952 bp, as expected for a genome belonging to the *ovis* biovar (Table 4). The same CAC motif can be observed in the lineage *C. pseudotuberculosis* MB154 at coordinates 65124–65135 bp, as expected for a strain of the *equi* biovar. Since the same motif can be located in different regions between biovars, these microsatellites showed potential as biomarkers capable of differentiating biovars.

Statistical analysis showed that the two groups of microsatellites belonging to *equi* and *ovis* biovars are similar, thus indicating that they are good candidates for markers for *C. pseudotuberculosis*. It was still possible to observe a feature difference in position 3 of SSR patterns between the two groups (Figure 6) and a difference between the *equi* and *ovis* biovars, thus indicating the possibility of using them as biovar markers as well.

As a zoonotic bacterial pathogen, *C. pseudotuberculosis* is widely spread bacteria that infect many kinds of animals; however, biovar differentiation remains to be a challenging task (Almeida et al., 2017). The groups of SSR patterns identified in this work could serve as an *in silico* alternative and could be employed as potential biovar-specific biomarkers for *C. pseudotuberculosis*. In addition, the correct diagnosis and identification of many other major bacterial pathogens also impose a great challenge to public health and veterinary practice worldwide. Therefore, the computational methodology applied to this issue here could also be applied to other bacterial pathogens in the future.

## Conclusion

Different patterns of microsatellites, or simple short repeats (SSRs), were observed for different strain groups of *Corynebacterium pseudotuberculosis*, and SSRs unique to the strains and distinct from the other genomes were evaluated in this study. Patterns of SSRs associated with genes that encode virulence factors (VFs) were also identified, being all of the SSR motifs related to the same SpaH-like pili VF in all genomes. These VR-related SSRs can serve as indicators of the genome organization and identify polymorphisms among the strains evaluated here.

In addition, microsatellites are important evolutionary markers and can be isolated by NGS technology from the genome of a model and non-model species, allowing the tracking of SSR length variations, such as point mutations and duplications across the entire genome to identify similarities and differences among strains.

Finally, the results in this work demonstrated an unexplored potential for using these molecular markers not only for the identification of species and strains but also in the screening of specific biovars. Therefore, the study of SSRs has been proven crucial to the understanding of the genomic content, dynamics, and structure of bacterial pathogens, such as *C. pseudotuberculosis*.

## Data Availability

Publicly available datasets were analyzed in this study. These data can be found at: https://www.ncbi.nlm.nih.gov/refseq/.

## Abbreviations

ML, maximum likelihood; NCBI, National Center for Biotechnology Information; NGS, next-generation sequencing; NJ, neighbour-joining; SSRs, simple short repeats or microsatellites; VFs, virulence factors.
