# Supplementary figures and images for: In silico approach to identify microsatellite candidate biomarkers to differentiate the biovar of Corynebacterium pseudotuberculosis genomes
Source: Front Bioinform. 2022 Sep 16;2:931583. doi: 10.3389/fbinf.2022.931583 (PMC9580864; doi:10.3389/fbinf.2022.931583)

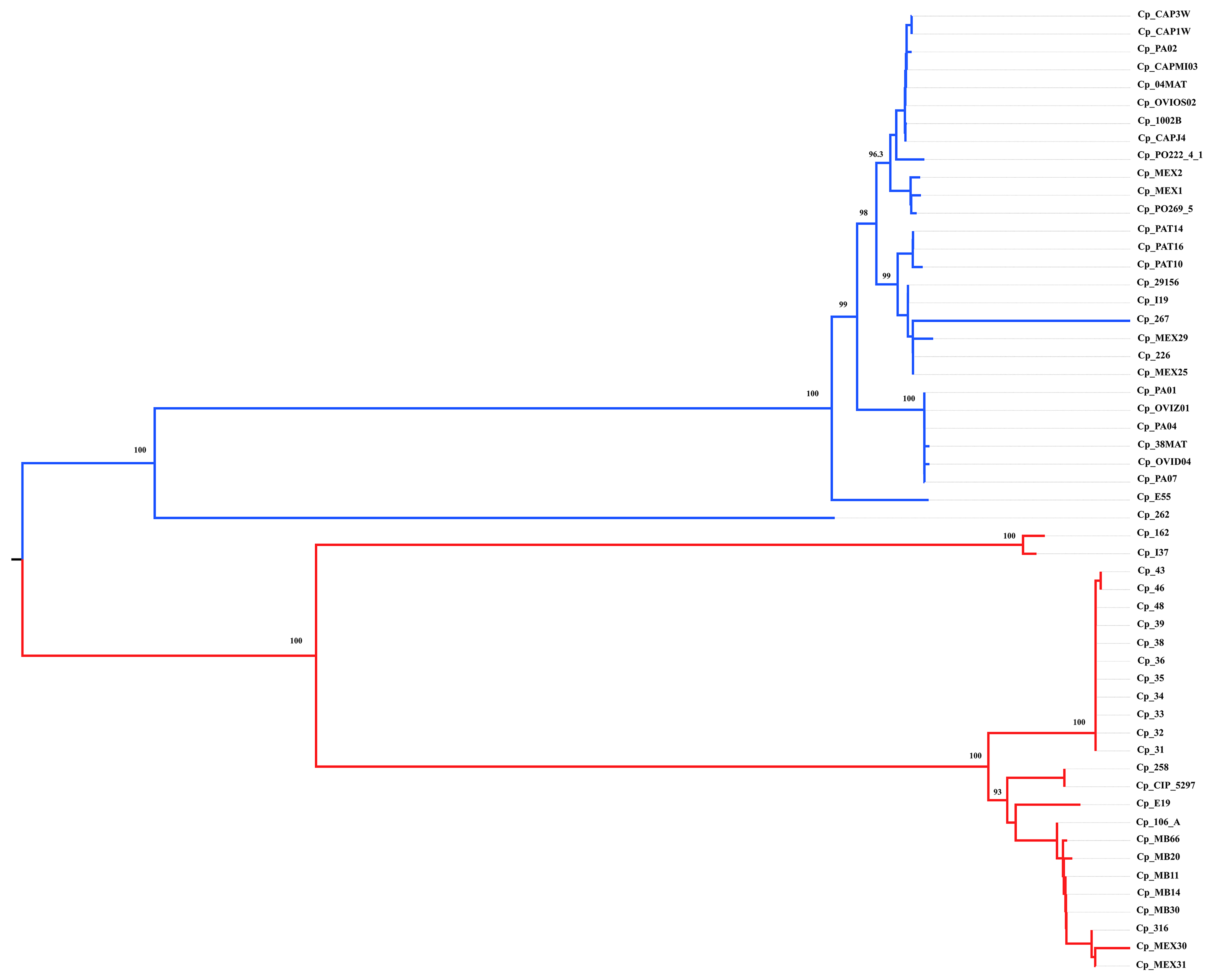

Supplement: Supplementary file 3 [file Image1.TIF]
